# Universal model for the skin colouration patterns of neotropical catfishes of the genus *Pseudoplatystoma*

**DOI:** 10.1038/s41598-020-68700-0

**Published:** 2020-07-24

**Authors:** Pablo Scarabotti, Tzipe Govezensky, Pablo Bolcatto, Rafael A. Barrio

**Affiliations:** 1 Instituto Nacional de Limnología, UNL, CONICET, FHUC, Ruta 168 Km 0, Ciudad Universitaria, S3001XAI Santa Fe, Argentina; 2 Instituto de Investigaciones Biomédicas, Universidad Nacional Autónoma de México, 04510 CD.MX., Mexico; 3 Instituto de Matemática Aplicada del Litoral, UNL, CONICET, FHUC, IMAL, Colectora Ruta Nac. 168 km 0, Paraje El Pozo, S3007ABA Santa Fe, Argentina; 4 Instituto de Física, U.N.A.M., Apdo. Postal 20-36, 01000 CD.MX., Mexico

**Keywords:** Applied mathematics, Morphogenesis

## Abstract

Fish skin colouration has been widely studied because it involves a variety of processes that are important to the broad field of the developmental biology. Mathematical modelling of fish skin patterning first predicted the existence of morphogens and helped to elucidate the mechanisms of pattern formation. The catfishes of the genus *Pseudoplatystoma* offer a good biological study model, since its species exhibit the most spectacular and amazing variations of colour patterns on the skin. They present labyrinths, closed loops (or cells), alternate spots and stripes, only spots and combinations of these. We have extended a well known mathematical model to study the skin of *Pseudoplatystoma*. The basic model is a two component, non-linear reaction diffusion system that presents a richness of bifurcations. The extended model assumes that there are two interacting cell/tissue layers in which morphogens diffuse and interact giving rise to the skin colouration pattern. We have found that by varying only two parameters we are able to accurately reproduce the distinct patterns found in all species of *Pseudoplatystoma*. The histological analysis of skin samples of two species of this genus, with different patterns, revealed differences on the disposition of the colouration cells that are consistent with our theoretical predictions.

## Introduction

The outstanding diversity of fish skin colour patterns has fascinated scientists from a wide range of disciplines for a long time^1–4^. Skin pigmentation has important biological functions, preventing harmful radiation from damaging vital tissues; and complex colouration patterns can act as signals in social behaviour, camouflage, mimicry, and sexual display^4^. Fish skin colouration has been used to model mechanisms of pattern determination^5, 6^. This interest is partly due to the variety of processes that are important in forming pigmentation patterns, with unique characteristics during the development and growth of animals^7^. It is only recently that the development and evolution of pigmentation in fishes is in the process of being understood^4^.

The ontogenic origin of colouration patterns in fishes is largely determined by short range and long range interactions of three different cell types: melanophores, iridophores, and xanthophores^3, 7, 8^. Usually these cells are distributed in superimposed monolayers. The progenitors of these cell type originate at the neural crest and migrate to the fish skin through peripheral nerves, predominantly over the dorsal and ventral myotomes and along the horizontal myoseptum (the structure separating the dorsal and ventral halves of the myotome) to set up a three longitudinal line larval pre-pattern from which the adult pattern develops^9^.

Variation in colouration patterns between species may be due to genetic differences^3, 10^ or environmental influences^11^. For example, stripe patterning in zebra fish seems to be largely determined by genetics. Allelic variations on a single gene can produce a full range of patterns from stripes to small spots. The study of the origin of a specific variation in colouration pattern (or any other trait) can be accomplished by the comparison of the ontogenetic origin of that characteristic among a group of related species^9, 12^. Studying phenotypic and ontogenetic differences among closely related species can help elucidate the underlying mechanisms producing this variation, because related species share many genetic and developmental pathways, like mutants of an individual species.

Reaction–diffusion and related models have been applied to simulate pattern formation in several fish species^2, 6, 7, 13, 14^. Single component models predict simple patterns, as in the zebra fish^13^. Two-component Turing systems predict a wide variety of patterns of dots, stripes and combinations of them, as the ones in *Pomacanthus imperator* and other fish^14^. Pattern variation between spots and stripes can be predicted by simulations of a Turing model, by changing the value of the ratio between cubic and quadratic non-linearities in the kinetics^15^.

A good biological model to be described with reaction–diffusion systems are the shovel-nose catfishes of the genus *Pseudoplatystoma* (commonly known as Surubí or Surubim) that contains different species which are limited to South America, inhabiting the main river basins of the subcontinent (Amazon, Orinoco, Paraná, San Francisco and Magdalena)^16^. They are large migratory predatory fishes^17, 18^ and play an important ecological role in top-down regulation^19, 20^. Furthermore, in all the regions where they dwell, these catfishes are subject to intense commercial and sport fishing^21^.

**Figure 1:**
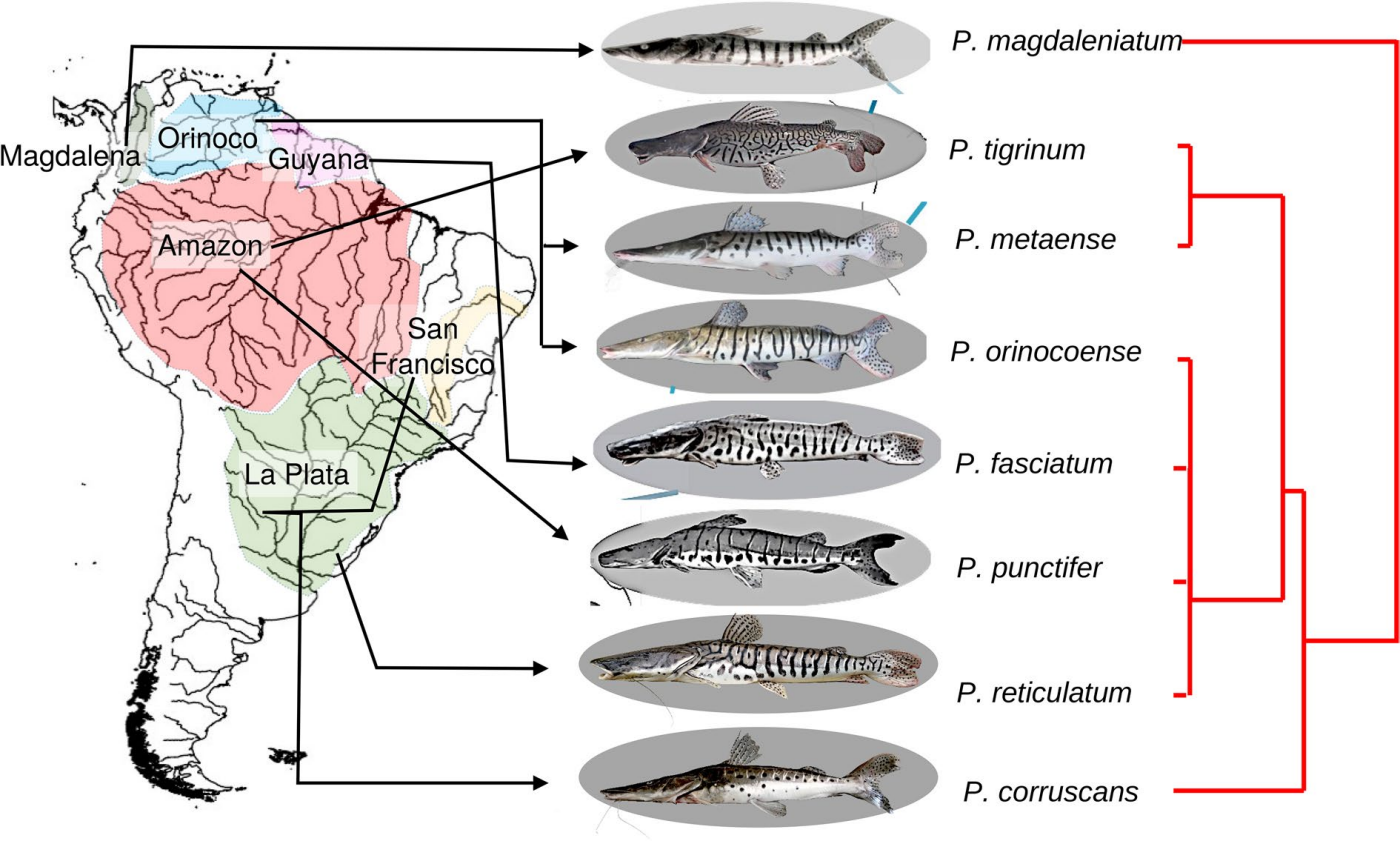
Geographical distribution of the eight species of the genus *Pseudoplatystoma*^22^ and their phylogenetic relationships (red lines following Ref.^23^). Photos of *P. fasciatum, P. magdaleniatum, P. metaense, P. oronocoense, P. punctifer, P. tigrinum* extracted of www.fishbase.se (access on April, 2020). Photos of *P. corruscans and P. reticulatum* by *P. Scarabotti*.

Currently, there are eight recognised species of *Pseudoplatystoma*:^22^ *P. orinocoense, P. punctifer, P. fasciatum, P. reticulatum, P. corruscans, P. magdaleniatum, P. tigrinum* and *P. metaense*. However, more recent genetic studies^23^ suggest the presence of four main taxonomic units throughout South America. All species develop complex skin colour patterns including spots, stripes, labyrinths, cells, and combinations of them (Fig. 1). Colouration patterns have been used in the definition of several species, but this trait seems to be extremely polymorphic within each species.

It is our aim to propose a universal mathematical model that suggests that there is a single mechanism for the patterning of the skin in this genus, and that the evident and enormous differences between them are due to a simple interaction between layers of cells located just below the epidermis near the *basal membrane*, and the abundance of one morphogen in the outer layer. In order to do that we need to take into account several important biological facts that are described in the next section.

**Figure 2:**
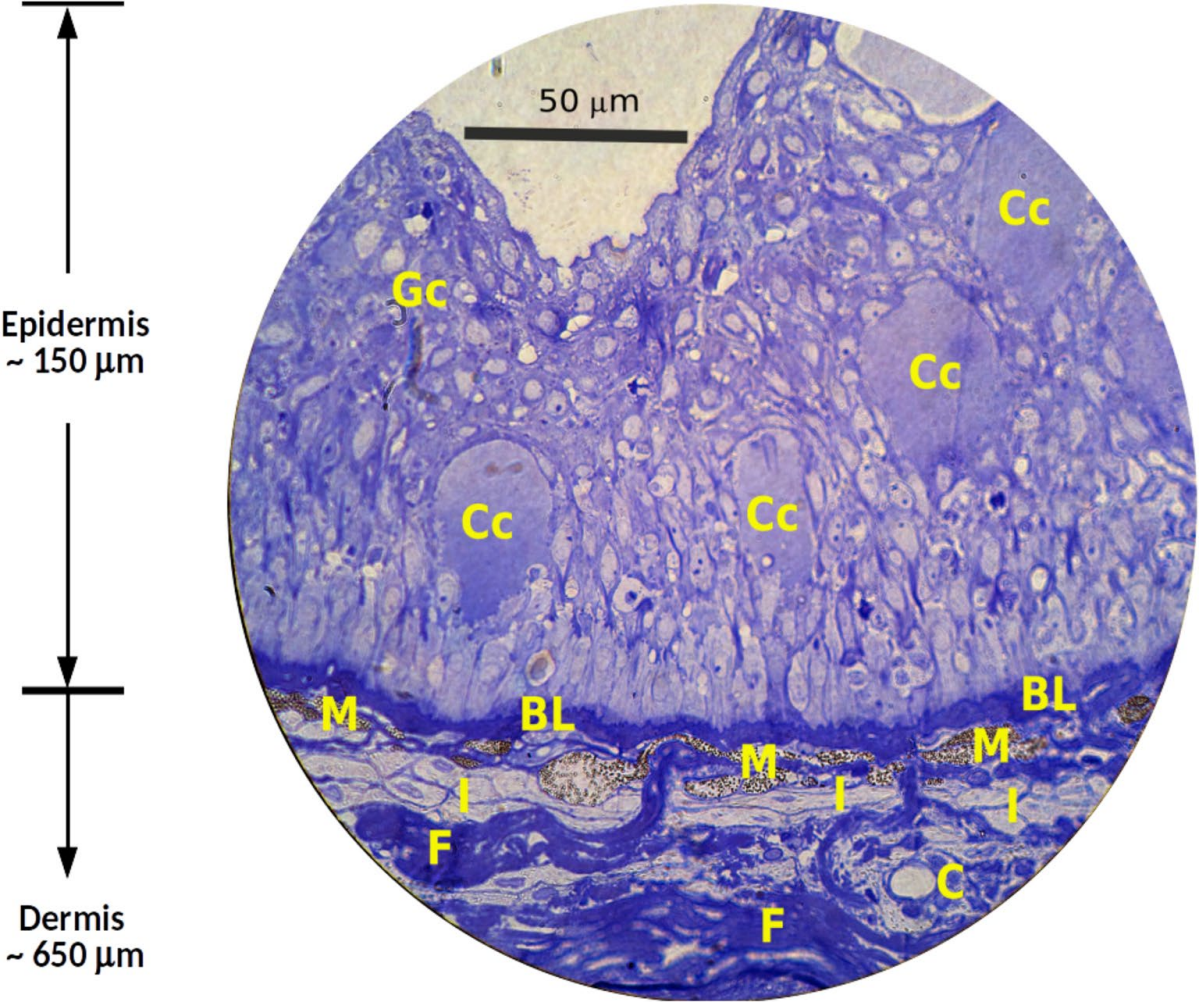
Histological cut (cross section) of the typical skin of *Pseudoplatystoma*. BL: basal epithelial cell layer (basal lamina). C: capilar. Cc: club cells. F: fibroblasts. Gc: goblet (mucus) cells. I: iridophores. M: melanophores.

## Results

### Histological description of the skin of *Pseudoplatystoma*

The structure of the skin of *Pseudoplatystoma* species (see Fig. 2) is similar to the skin of other catfishes^24^ and consists of two main layers: the superficial epidermis and the underlying dermis (Fig. 2). The epidermis has a variable thickness between 100 and 200 $\upmu$m. The bottom of the epidermis is formed by a monolayer of columnar germinative cells from which the epidermal cell types grow and differentiate: the basal lamina.

The largest and most conspicuous cells in the epidermis are the club cells (Cc), associated with immune response and alarm reaction to predators^25^. These cells are oval, reaching 40–50 $\upmu$m in length and are placed in the middle layer of the epidermis. The goblet or mucus cells (Gc) are small polyhedral cells with a clear cytoplasm, that are distributed in the uppermost layer of the epidermis. These cells migrate to the surface and release their mucus content forming the pore goblet cells. The remaining spaces of the epidermis are filled by epidermal epithelium cells. There are no chromatic cells in the epidermis of *Pseudoplatystoma*.

The dermis is a layer of clear tissue of 600–700 $\upmu$m thickness, largely composed of fibroblasts (F) and joined to the epidermis by the basal lamina (BL), an undulated layer of extracellular fibrous material of 2–7 $\upmu$m thick.

The chromatophores, which are the cells that give the colouration to the skin, are placed on the upper portion of the dermis (50 $\upmu$m thick), just below the basal lamina. At least two types of chromatophores can be distinguished in the skin of *Pseudoplatystoma*: melanophores (M), that are large and flattened dendritic cells containing numerous black vesicles of melanin; and iridophores (I), that are smaller and flattened cells containing small whitish polyhedral guanine crystals in their cytoplasm.

### Description of the model

The well established BAVM model (after Barrio, Aragon, Varea and Maini)^14^ is the most general reaction–diffusion system with quadratic and cubic non-linearities that conserves mass. It presents a wide variety of bifurcations (Turing, Turing-Hopf, saddle-node and excitable media, chaos^27^) due to the existence of three fixed points, allowing a great variety of spatial patterns. This model has been used previously to describe the colouration pattern of Surubim^28^. However, the authors used a single reaction–diffusion system, which was not versatile enough to reproduce all the observed patterns in the genus, even by using different aligning boundary conditions, space dependent diffusion coefficients and additional advection terms. In this work we show that it is possible to describe the pattern formation mechanism by using the BVAM model in a unified and consistent way by taking into account the biological characteristics of the skin of the fish.

Considering the structure of the skin of *Pseudoplatystoma* and the positioning of the chromatic cells in superimposed layers, we need to construct at least two coupled reaction diffusion systems that simulate two different layers of the skin in which the morphogens react.

Reaction–diffusion systems deal with the dynamics of concentrations of morphogens, and it would be misleading to consider that morphogens are the only factor that determines cell fate, which ultimately result in the pattern observed on the skin. There is a vast literature dealing with the mechanisms by which cells receive and process the information given by the morphogens to determine differentiation and motility (see^29^ and references therein), which we will not address here. From now on, we will make the assumption that the migration and reproduction of chromatophores follows the variations of the concentrations of morphogens that forms a pattern, as it seems to be the case in other systems modelled by reaction–diffusion models^6, 7^.

In a previous work^26^ we proposed a model coupling two reaction–diffusion systems that was able to simulate the variation in colouration patterns in different species of stingrays of the genus *Potamotrygon*. The essential feature of that model was that we used one system tuned in the Turing region to describe the density of mesenchymal cells and extracellular matrix (dermis), and another system to describe the overlying epidermis, where the colouration is apparent. The two layers are coupled (either linearly or cubically) via interaction terms between the systems.

There are important differences between that model and the one we are proposing here. First, the patterns of *Pseudoplatystoma* species evidently are not of the Turing type, since they do not conserve the spatial scale while the fish grows (as in *Pomacanthus*), a distinct property of Turing patterns. The other difference is that the fish exhibit thin vertical whitish lines distributed serially on the flanks of the fish, that seem to orient the patterns vertically in some species.

The model we propose reads,

1a $$\begin{aligned} \frac{\partial u_1}{\partial t}&= D_1 \nabla ^2 u_1 + \eta _1 \left( u_1 + a_1v_1 - C_1u_1v_1 - u_1v_1^2 \right) +q(u_2-u_1), \end{aligned}$$

1b $$\begin{aligned} \frac{\partial v_1}{\partial t}&= \nabla ^2 v_1 + \eta _1 \left( b_1v_1 + h_1u_1 + C_1u_1v_1 + u_1v_1^2 \right) +q(v_2-v_1),\end{aligned}$$

1c $$\begin{aligned} \frac{\partial u_2}{\partial t}&= D_2 \nabla _2^2 u_2 + \eta _2 \left( u_2 + a_2v_2 - C_2u_2v_2 - u_2v_2^2 \right) -q(u_2-u_1),\end{aligned}$$

1d $$\begin{aligned} \frac{\partial v_2}{\partial t}&= \nabla ^2 v_2 + \eta _2 \left( b_2v_2 + h_2u_2 + C_2u_2v_2 + u_2v_2^2 \right) -q(v_2-v_1), \end{aligned}$$

where *q* is the linear interaction parameter between the two models, whose variables are labelled with sub-indices $i=1$ (inner layer) and $i=2$ (outer layer). Here, $u_{i}$ and $v_{i}$ represent relative abundance of chemical morphogens, $D_{i}$ is the ratio of the diffusion coefficients $D_{u_{i}}/D_{v_{i}}$. The constants $a_{i}, b_{i}, C_{i}$ and $h_{i}$ ($i=1,2$) are the parameters of the kinetics, and $\eta _{i}$ gives the size of the system. Observe that the layers are supposed to be flat and on top of each other, so the interaction terms between layer takes place in the direction perpendicular to the planes, that is $q(u_2-u_1)=q(u_2(x,y)-u_1(x,y))$.

**Table 1:** Values of the parameters of the BVAM system used to reproduce the patterns resembling all species of Surubim.

Parameter	Value
$a_1=a_2$	0.05775
$b_1=b_2$	$-$ 0.30525
$h_1=h_2$	$-$ 1.30000
$C_1=C_2$	0.02000
$\eta _1=\eta _2$	0.47000
$D_1=D_2$	0.46600

We take advantage of the richness of bifurcations exhibited by this model to choose parameters that produce wave fronts, whose velocity is regulated by the parameters *C* and *h* so they could be arrested. In principle there is no reason to assume that diffusion of morphogens and the parameters of the systems should be different in the two layers, except probably for the time scale of the dynamics and the initial disposition and abundance of morphogens in the two layers.

In order to test these assumptions we performed numerical calculations using the values shown in Table 1. We used a simple Euler method to integrate Eq. 1 with a time step of $\delta t=0.01$ and found reliable convergence for 240,000 time steps. In all the simulations, we used initial conditions of the form $u_0+\zeta$ and $v=\zeta$, where $\zeta$ is a random number from a flat distribution of width 0.02 and zero mean. Then, the parameter $u_0$ could be identified as the actual concentration of morphogen in the fish, which could vary according to the species.

We observed that the three basic geometric motifs observed among the species, namely, cells, stripes with round heads and spots (not necessarily circular), are present in this calculations, and their proportion varies according to the initial value of the morphogen $u_0$. We propose that this parameter can vary within as well as between species, since the patterns vary widely among individuals of the same species. However, there is no orientation in these patterns. In order to get it, we introduced a pre-pattern based on the ontogenic origin of colouration.

### Simulation of oriented patterns

In most of the species, periodic stripes are more or less oriented vertically and the orientation of the stripes may well depend on the environmental cues mediated by underlying tissues^3^, as suggested by the vertical white lines mentioned before and quite apparent in Figs. 1 and 6.

**Figure 3:**
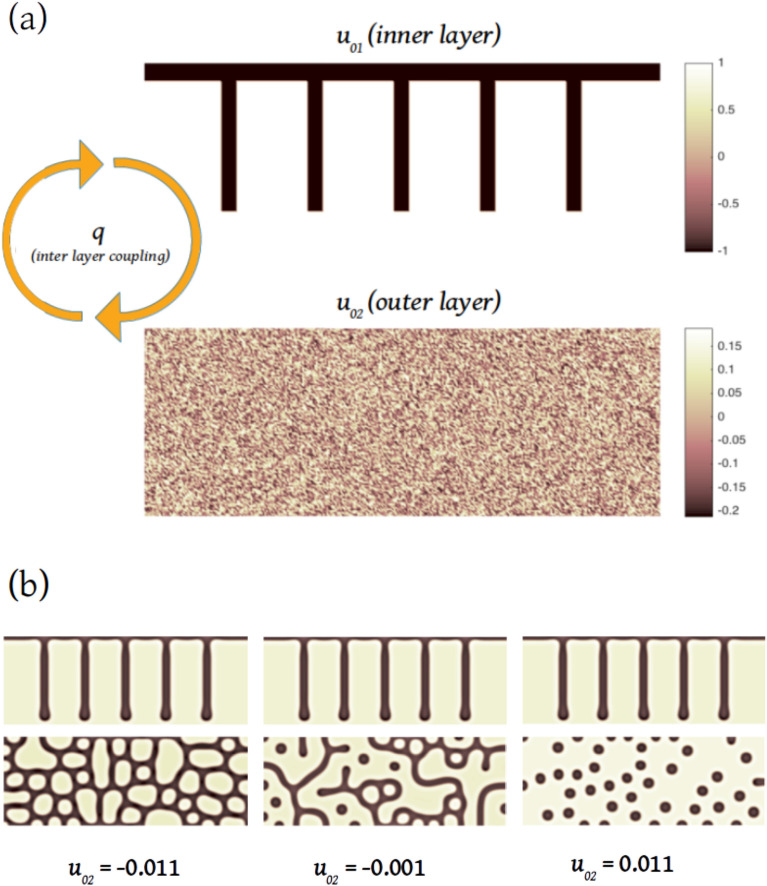
(**a**) Scheme of the initial condition for the calculations. The morphogens are randomly seeded at the outer layer and they are driven by the comb-like distribution at the inner layer through the coupling *q*. (**b**) Examples of calculations made using the pre-pattern in the inner layer and random initial conditions for the outer layer. We show three values of $u_{02}$ for $q=0$. Observe the transition from cells to dots in the outer layer.

We have considered two morphogens that can react and diffuse inside and between the inner and outer layers. The variables *u* and *v* in this context represent the densities of morphogens measured from an average value, so that they will assume positive/negative values when there is an excess/deficiency of morphogen in a particular region of the skin.

The domain size simulates a half of the dorsal part of the fish body and we assume that the dermis is divided by anatomic features that could disturb the spatial equilibrium density of the morphogens. In order to take this into account we assume that there is an accumulation of $u_1$ (or $v_1$) on the top line and on vertical stripes. Consequently, the initial conditions for $u_{01}$ are set to 1 everywhere and − 1 in the stripes in a comb-like pre-pattern, as shown in Fig. 3a. On the outer layer we use random initial conditions for $u_{2}$ around a given level of morphogen $u_{02}$. As the simulation domain resembles the finite length of the fish, we impose Neumann or zero flux boundary conditions, i.e. the gradient of concentrations at the boundaries is nil.

Examples of calculations made under these conditions are shown in Fig. 3b. Observe that there is a transition from cells to stripes and dots when varying $u_{02}$.

**Figure 4:**
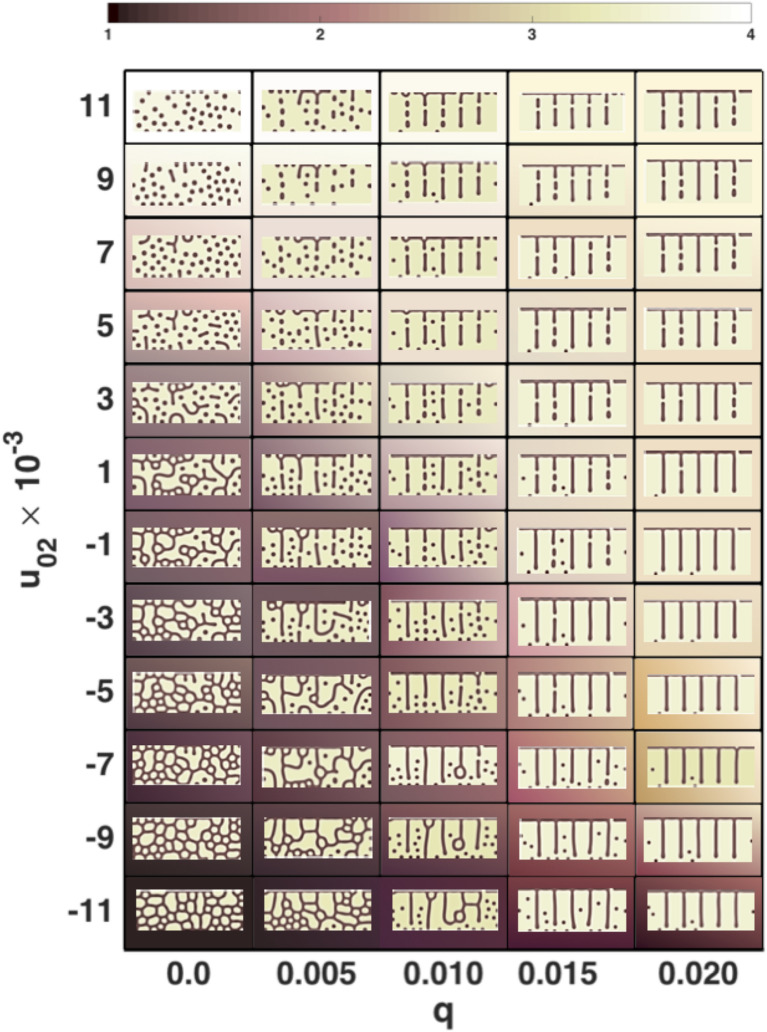
Phase diagram showing regions of different steady patterns of $u_2$ on the outer layer, obtained with numerical calculations using the same initial random conditions. The colour bar on the top indicates smooth transitions between regions in which one distinguishes patterns with (**1**) only cells, (**2**) cells, dots and stripes, (**3**) dots and stripes and (**4**) only dots.

With these preliminary calculations, we explored patterns obtained by varying only $u_{02}$ and *q* and predicted the values needed to obtain the patterns of all the species.

In Fig. 4 we show a phase diagram in the $[u_{02},q]$ space. Observe that the variations of *q* are very small, which is to be expected if these variations occur even within a single species. Four different qualitative regions can be identified (which are correlated with the background-colour palette). (1) Is a region mainly with cells and sparse labyrinths, (2) a complex region where spots appear, cells have minor occurrence meanwhile labyrinths remain, (3) is a mix of stripes and dots and, (4) the simplest region in which almost only spots (not necessarily circular) are present. Observe the smooth transition between these regions.

**Figure 5:**
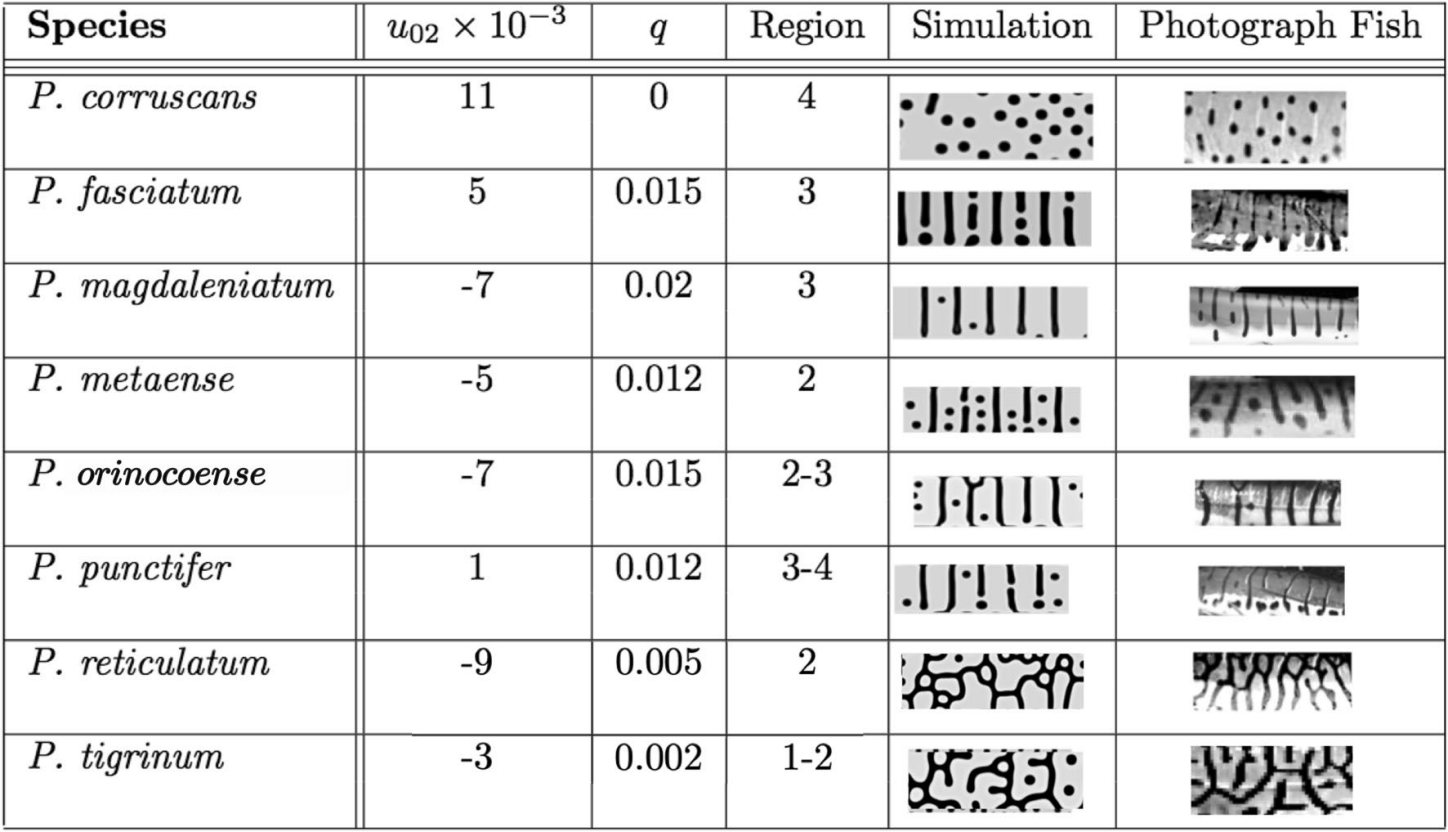
Simulated patterns (fifth column) that could be compared to the observed skin patterns of the different species of *Pseudoplatystoma* (right hand side column). We can obtain all the key geometrical patterns for all species in the first column, as stripes, spots and cells by maintaining all the parameters fixed and just varying the initial amount of morphogen $u_{02}$ and the coupling *q* between the two linearly coupled BVAM systems.

Due to the richness of the different patterns obtained as final results of the simulations, it is possible to identify the typical patterns of each species of *Pseudoplatystoma*. In Fig. 5 we show the values of the parameters that best capture the main features of each pattern, and a comparison between the numerical patterns and the skin of the fish. The similarity between observed skin colouration and the simulated patterns is remarkable.

**Figure 6:**
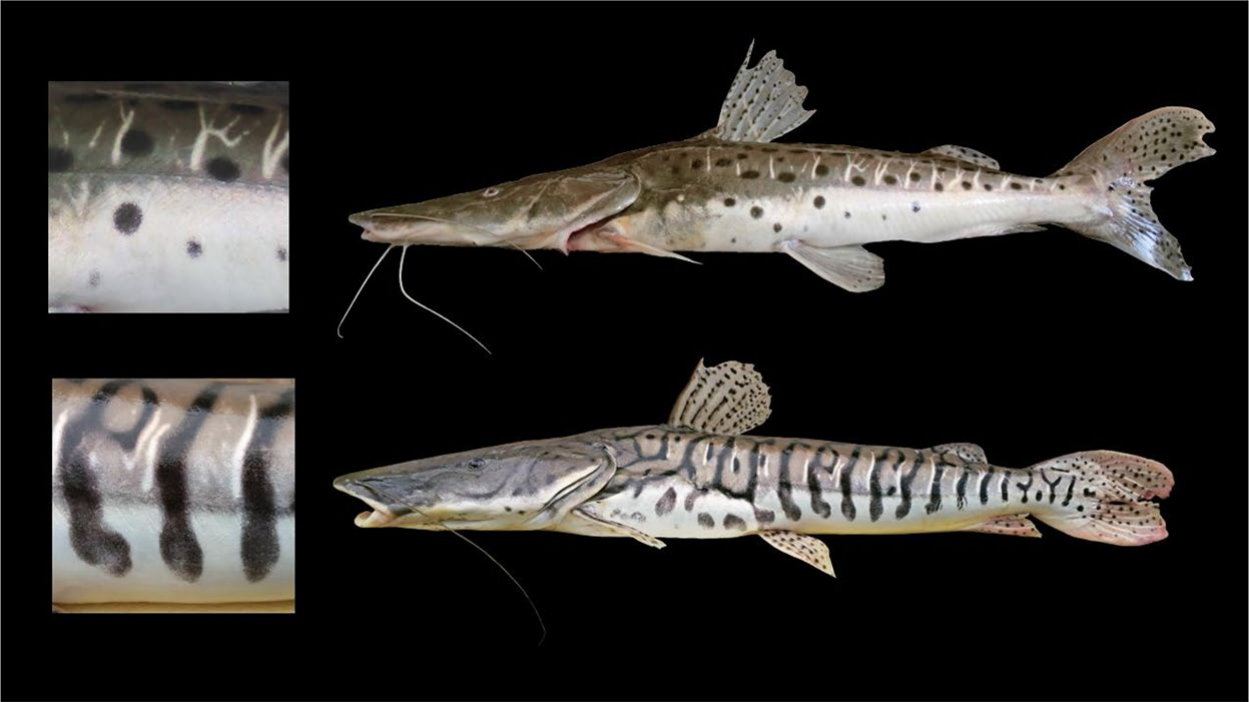
Photographs of two species of *Pseudoplatystoma* with contrasting colouration pattern collected at the same locality: *P. corruscans* (above) and *P. reticulatum* (below). Magnifications on the left show the details of the colouration pattern. Notice the dorso-ventral counter-shading, black spots and stripes, and the vertical whitish lines that compartmentalise the pattern.

### Experimental validation of the model

*Pseudoplatystoma corruscans* and *Pseudoplatystoma reticulatum* are both found in the same region of the Paraná river. They share the same environmental conditions, food resources, climate, exposure to predators, etc. However the differences in the colouration pattern of their skins are very noticeable. *P. corruscans* have a pure spotted pattern (region 4 of the phase diagram) and *P. reticulatum* have stripes, cells and labyrinths (regions 1–2 of the phase diagram). Photographs of the two species are shown in Fig. 6. We have explored the foundations of our hypotheses by studying specimens of these two species with very different colouration patterns but sharing the same environments. Skin samples were obtained from fresh specimens collected by fishermen in the Paraná River near Santa Fe city.

Fish exhibit two distinct adult pigment patterning mechanisms: dorso-ventral counter-shading patterning and a distinct colouration pattern. These are largely independent of each other, superimposing themselves to give the resulting full colouration^30^.

The genus *Pseudoplatystoma* has a characteristic colouration pattern consisting in black stripes or spots that extends dorsolaterally on a grey background colouration in the dorsal half of the fish, and a white background colouration in the ventral half (see Fig. 6). In the spots and dorsolateral stripes, the melanophores are arranged in the uppermost layers of the dermis, giving origin to the dark colouration, while the iridophores are arranged beneath, probably increasing the brightness of dark sectors. In the dorsal background colouration, that usually show a metallic grey colouration, a central layer of melanophores is flanked by an upper and lower layer of iridophores. These iridophores could disperse the light in different ways according to the orientation of the guanine crystals, and interacting with the dispersion of the melanophores^31, 32^, can generate a diversity of background hues.

In addition to black stripes and spots, the skin pattern of *Pseudoplatystoma* is characterised by the presence of thin white vertical lines (which sometimes have a dendritic configuration), which occur in a number of 10–15 in the lateral area of the fish. In all *Pseudoplatystoma* species, the formation of a clear horizontal mid line in the horizontal myoseptum is observed^33, 34^, which would form the main pre-pattern in similar way to what occurs in the zebra fish^8^. These vertical lines begin to grow from this clear horizontal bar in dorsal direction, and become evident when the fish reach 10 or 15 cm. The white lines are rarely crossed by spots and black stripes, presumably functioning as effective delimiters of the lateral expansion of the lines and black spots (see detail in Fig. 6). These lines are present in all species of *Pseudoplatystoma*, except *P. tigrinum* and *P. metaense*, in which they are imperceptible or absent, and colouration pattern has a greater presence of diagonal and horizontal dark stripes^22^, which reinforces the hypothesis about the role of vertical lines in the horizontal compartmentalisation of the pattern. Histologically, the only chromatic cells present in these vertical lines are iridophores.

**Figure 7:**
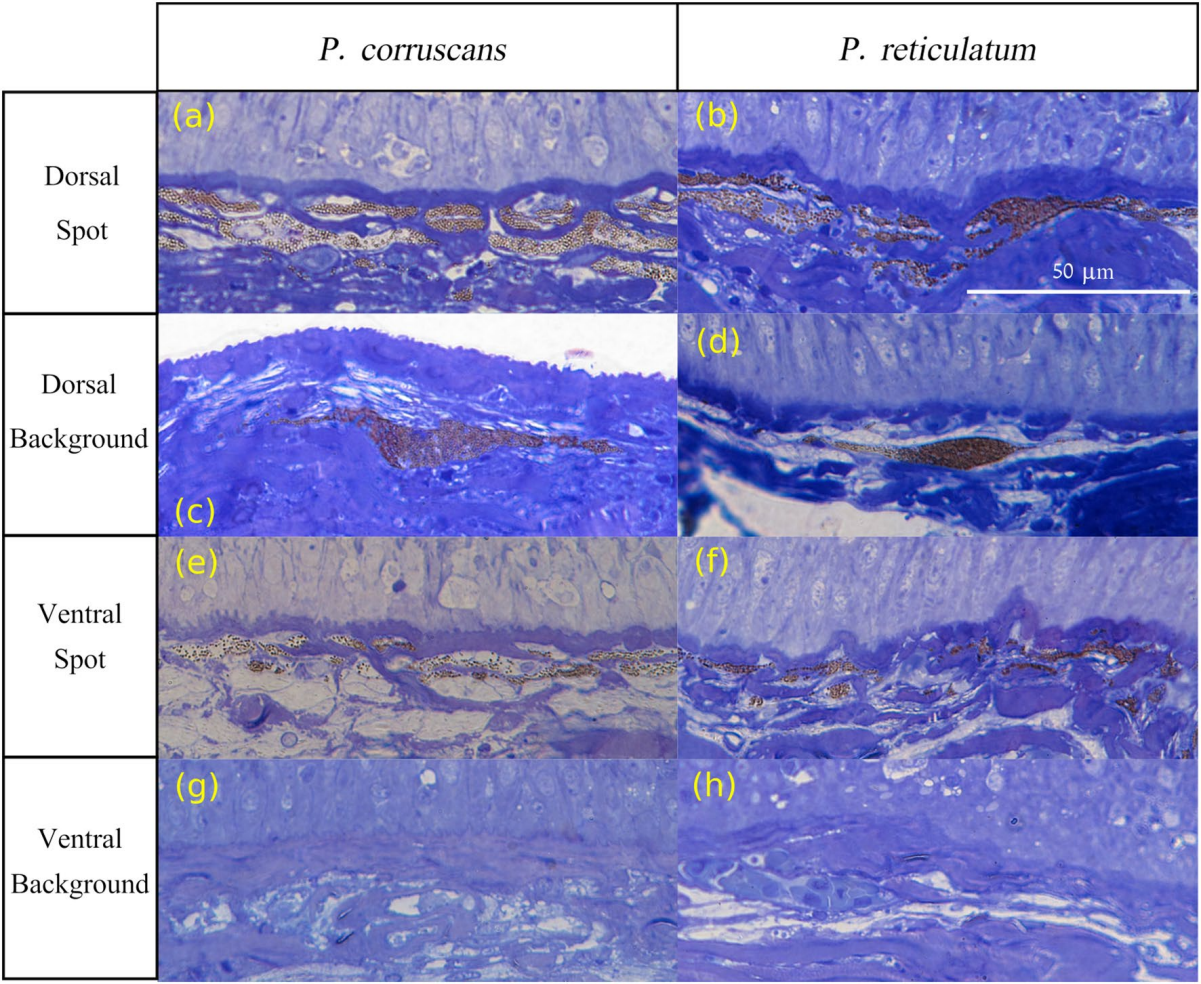
Histological sections of dorsal and ventral skin of *P. corruscans* (left panel) and *P. reticulatum* (right panel). (**a**,**b**) Dorsal spot. (**c**,**d**) Dorsal background. (**e**,**f**) Ventral spot. (**g**,**h**) Ventral background.

In Fig. 7 we show histological sections of four different significant places of the skin of both species. The first row are images of the spots on the dorsal region, the second one corresponds to the background of the dorsal region where the spots are not present, the third row shows cross-views of the spots of the ventral region (not included in the simulations) and in the last row there are images of the white skin of the belly. As indicated in Fig. 2 the two main chromatic cells are melanophores (that contain dark circular vesicles) and iridophores (that contain clear stick-shaped crystals) .

The histological sections of the skin showed that the sites with the greatest proliferation of iridophores (where the layer of iridophores is thicker), such as the ventral zone and the background colouration of the dorsal area, are coincident with areas with less proliferation of melanophores and vice versa. This observation is consistent with a process of inhibition between iridophores and melanophores, as observed in zebra fish^7, 8^

As the hypothesis of the model predicts, the major density of melanophores is located on the patterned zone inside the spots or stripes (Fig. 7a,b). These chromatic cells forming the pattern are separated by an intermediate layer of iridophores so that two layers of melanophores can be distinguished, although the layers are not completely smooth and regular. In *P. reticulatum* these two layers eventually collapse, which can be interpreted as an indicator that the two layers are coupled by a real interaction that causes the migration of melanophores after the removal of iridophores, as predicted in the model calculations.

The collapse of the layers is not present in *P. corruscans*, which also enforces the theoretical result giving only dots with $q=0$ (zero coupling in region 4 of Fig. 4). In the background colouration of the dorsal region (Fig. 7c,d), the situation is clearly different. There is only one layer of melanophores underneath a layer of iridophores that mask them, reflecting the outside light and preventing the visualisation of a pattern on the skin. The images of the ventral spots (Fig. 7e,f) are quite similar to the dorsal ones but with a significantly lower density of melanophores. In the black spots of the ventral portion of *P. reticulatum* one notices one or two almost continuous layers of melanophores that are placed above the iridophores which in turn are disposed in two to four layers.

Finally, on the white zone of the belly (Fig. 7g,h) only iridophores are present. Iridophores can be present in one to four discontinuous layers of up to 12 $\upmu$m thick (layers can be compact or loose) and are always separated from the basal lamina (or from the epidermis) by one or more layers of fibroblasts.

Melanophores and iridophores vary in abundance and position between the ventral and dorsal portion of the fish’s body and between black spots and stripes and the background colouration. Iridophores seem to be present in all parts of the body and melanophores are present only in the dark parts of the body (black stripes, spots and in the grey-brown dorsal background colouration) of the two species examined: *P. corruscans* (spots) and *P. reticulatum* (stripes).

### Discussion

In this work we have observed that the differences in the colouration pattern of the two species of *Pseudoplatystoma* observed under the microscope is determined by the differential distribution of layers of melanophores and iridophores at variable depths of the dermis, as observed in other fish species^35, 36^. To our knowledge, the iridophores had not previously been reported in any species of Siluriformes (see^24^), so this report expands the number of cell types that participate in the formation of skin colouration patterns in this group of fishes. The histological sections of the skin showed that the sites with the greatest proliferation of iridiophores (where the layer of iridophores is thicker), are coincident with areas with less proliferation of melanophores and vice versa. This observation is consistent with a process of inhibition between iridophores and melanophores, as observed in zebra fish^7, 8^. Iridophores inhibit the proliferation of melanophores in immediate areas, while stimulating their proliferation in neighbouring areas^4, 7^. This process, called local activation and long range inhibition, is the basis for the “wave” propagation process necessary for the formation of colouration patterns in fish^6, 37^. In the skin of *Pseudoplatystoma*, iridophores could play a regulatory role on the proliferation of melanophores during the formation of colouration patterns.

The mathematical model proposed in this work simulated the variation in the colouration pattern of all known species of *Pseudoplatystoma*, by only modifying the values of two key parameters: $u_0$, the concentration of the morphogen and *q*, the linear interaction/coupling between the two systems of the model. Firstly, *q* defines the influence of the pre-pattern present in the lower layer (not visible) on the appearance of the upper layer (visible). As the interaction between layers increases, the pre-pattern, composed of vertical stripes, increasingly delimits the horizontal development of the pattern of the upper layer. For instance, when the coupling is large, as in *P. magdaleniatum*, the vertical lines dominate, while in *P. tigrinum* (stripes and cells) the patterns are not oriented vertically, which is achieved by diminishing the coupling between layers. Consequently, one could infer that the function of the whitish vertical lines as horizontal delimiters of the pattern could be exercised by local inhibition and long range activation of the proliferation of melanophores^8^. This process would induce stripes to develop largely aligned and close to white lines, which in fact is the pattern observed in most *Pseudoplatystoma* species.

Secondly, the parameter $u_0$ determines the morphogen concentration and generates a change in the configuration of the pattern from spots to stripes when the interaction between layers in low. At high values of $u_0$, the model generates a pattern composed uniquely by random or evenly-spaced small circular spots, while at higher values there is a pattern formed exclusively by stripes, which can connect between them, forming cells or labyrinths. Mutations of a single gene called leopard (*leo*) can produce similar changes from spots, passing through irregular stripes, to parallel stripes, in a way very similar to that predicted by a reaction–diffusion model^6^. Molecular studies have determined that the *leo* gene codes for a membrane connexin that could be involved in cellular interactions and affect the motility and proliferation of chromatophores^38^. Mutants of this gene usually present reduced number of melanophores^38^, a characteristic that can be paralleled to the morphogen concentration in our reaction diffusion model. In *Pseudoplatystoma*, the $u_0$ parameter could be associated with the inhibition of the proliferation rate of melanophores. All species of *Pseudoplatystoma* have dots on their fins. Our model predicts that these patterns are observed either because in fins there is no pre-pattern or because the interaction between layers is null or very small. As the dotted pattern is generated at high morphogen concentrations and low coupling between layers, we predict a low melanophore proliferation in the fins of all species of *Pseudoplatystoma*.

We have shown that the variability of the colouration patterns on the skin of the fish *Pseudoplatystoma* can be explained with a general reaction–diffusion model in a system of two coupled layers. The mechanism producing the patterns here is quite different from the Turing instability observed in the Angel fish *Pomacanthus imperator*^14^, since in our equations the diffusion coefficients of all morphogens are the same and one is far from the Turing region. In here, the non-linear system exhibits a bistable situation that produces travelling waves whose velocity is regulated by the ratio between quadratic and cubic non-linearities (parameter *C*)^39^.

The numerical simulations of the colouration patterns of the species using these two key parameters, make possible to identify the colouration patterns of the different species in a two-dimensional phase diagram (Figs. 4 and 5) and to infer their correspondence with the known phylogenetic relationships between species. The most recent genetic studies^23^ suggest that the eight species of *Pseudoplatystoma* can be grouped into four main clades (see phylogenetic tree in Fig. 1). The most basal node separates *P. magdaleniatum* (the phylogenetically more distant species) from the remaining species of *Pseudoplatystoma*. The colouration patterns of the species of the above mentioned clades appear to be located in different regions of the phase diagram, corroborating the idea that the patterns simulated by the model relate to each other in a manner consistent with the known phylogenetic relationships.

Black vertical stripes and white thin lines appear to be the primitive character that is present in most species forming the baseline condition from which the colouration pattern of all known species could have evolved (including the most basal species *P. magdaleniatum*^22^). On the other hand, the pattern observed in *P. corruscans* is differentiated by a notable decrease in the concentration of the morphogen and a low interaction level between layers, that give rise to their characteristic spotted pattern. In the remaining clade, the four species with a reticulated pattern, present various combinations of interactions an morphogen levels, and in the species *P. tigrinum* and *P. metaense* (that lack white vertical lines) the reduction of the interaction between layers results in the dominance of oblique and horizontal dark stripes. Hybrid specimens of *P. corruscans* $\times$ *P. reticulatum* exhibit both the presence of stripes and spots, which places these specimens in an intermediate point between the patterns of the two parent species, giving an interesting study model to analyse the genetic control of the colouration pattern as in other fish species^10^.

These predictions seem to be consistent with the current phylogenetic arrangement^23^ and could contribute to an adequate use of the colouration pattern as a systematic character in the differentiation of the species^22^. Finally, the detailed and comparative analysis of the ontogenetic origin of the patterns of all species of the genus could shed light on the evolutionary relationships of the patterns and the mechanisms by which the colouration originates, as was done in other species^12^. We think that the model proposed here can be a guidance and great help in investigating the chemical (at molecular level), and biological (at cell level) processes that ultimately result in patterning.

## Methods

### Experimental setup

The histology of the skin was observed in specimens *P. corruscans* and *P. reticulatum* collected by fishermen in the Paraná River near Santa Fe city.

Specimens were euthanized by percussive stunning and posterior medullar denervation. The collection of specimens was authorised by the Bureau of Sustainable Management of Fishery Resources of the Santa Fe Province (Dirección Provincial de Manejo Sustentable de los Recursos Pesqueros) and the methods used were approved and performed in accordance with the guidelines and regulations of the Committee of laboratory Ethics and Security of the Consejo Nacional de Investigaciones Científicas y Técnicas (CONICET). Skin samples were obtained from fresh specimens. Samples of 2 $\times$ 2 mm were taken from four locations along the fish body: in the dorsolateral portion of the body (1) inside and (2) outside a spot; and in the ventrolateral portion of the body (3) inside and (4) outside a spot (Fig. 7). Tissue samples were conserved in 100 mM PO_4_ buffer pH 7.2 containing 4% formaldehyde and 2.5% glutaraldehyde at $4\,^\circ$C overnight. The samples were dehydrated in a graded series of ethanol/water concentrations; subsequently, a graded series of epon/araldite resin (Araldite 502/Embed 812 Kit, EMS) in propylene oxide was used for embedding^36^. Thin sections (2–3 $\upmu$m) were stained with toluidine blue. Stained tissue sections were examined with a Leica DM 3000 Led Microscope and images were obtained with a Leica DFC 7000 T camera at maximum resolution using the manufacturer’s software.

Figures of simulated colouration patterns were created using the software MATLAB, version R2020a (https://la.mathworks.com/products/matlab.html).
